# Paper Withdrawn by the Authors before the Issue Release

**DOI:** 10.3390/s121216250

**Published:** 2012-12-17

**Authors:** Shu-Kun Lin

**Affiliations:** MDPI AG, Postfach, CH-4005 Basel, Switzerland; Office: Kandererstrasse 25, 4057 Basel, Switzerland; E-Mail: lin@mdpi.com

The following paper: “Jung, S.; Kim, J.H.; Kim, S. Bloom Filter-Based Advanced Traceback Scheme in Wireless Sensor Networks. *Sensors***2012**, *12*, 16250–16261.” has been withdrawn at the request of the authors before the issue release of *Sensors* Volume 12, Issue 12. We apologize for any inconvenience this may cause.



