# Supplementary material for: Paper Withdrawn by the Authors before the Issue Release
Source: Sensors (Basel). 2012 Dec 17;12(12):16250–61. doi: 10.3390/s121216250 (PMC3571781; doi:10.3390/s121216250)
Supplement: Supplementary file 1 [file sensors-12-16250-s001.pdf]

Note:

The following paper:

Jung, S.; Kim, J.H.; Kim, S. Bloom Filter-Based Advanced Traceback Scheme in Wireless Sensor Networks. *Sensors* **2012**, *12*, 16250-16261.

was published on 23 November 2012. However, it was withdrawn by the authors on 17 December 2012 before the issue release of *Sensors* Volume 12, Issue 12.
